# Covid-19 associated ARDS in pregnant women and timing of delivery: a single center experience

**DOI:** 10.1186/s13054-022-04145-3

**Published:** 2022-09-13

**Authors:** Markus Busch, Marius M. Hoeper, Constantin von Kaisenberg, Thomas Stueber, Klaus Stahl

**Affiliations:** 1 Department of Gastroenterology, Hepatology and Endocrinology, Hannover Medical School, Carl-Neuberg-Str.1, 30625 Hannover, Germany; 2 Department of Respiratory Medicine, Hannover Medical School, Hannover, Germany; 3 German Centre for Lung Research, Hannover, Germany; 4 Department of Obstetrics, Gynecology and Reproductive Medicine, Hannover Medical School, Hannover, Germany; 5 Department of Anesthesiology and Intensive Care Medicine, Hannover Medical School, Hannover, Germany

## Dear Editor,

The SARS-CoV-2 pandemic resulted in an unprecedented number of severe cases among pregnant women [1, 2]. To date, there have been only few reports of the specific issues that arise during the intensive care treatment of pregnant women with lung failure due to Covid-19 [3, 4]. Complex medical decision-making is required in the management of critically ill pregnant women [5] and further data is needed to guide prognostication of outcomes and clinical decision making.

We here present a case series of 14 pregnant and peripartum women with severe acute respiratory distress syndrome (ARDS) due to Covid-19 treated at our institution between January 2020 and December 2021.

**Fig. 1:**
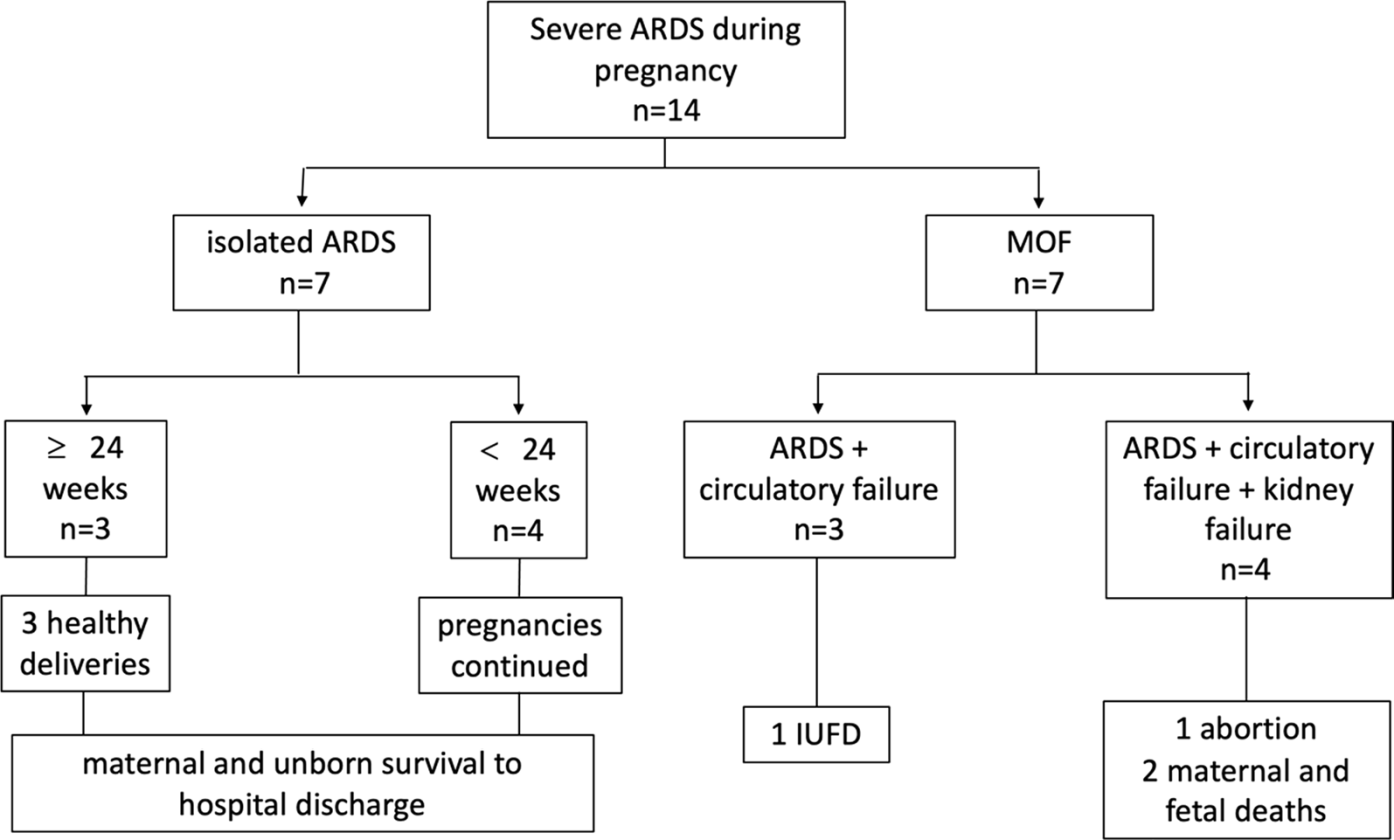
Diagram of the different ICU courses. Ad admission 7 patients had isolated ARDS and 7 patients had multiorgan failure (MOF). Three cesarean sections were performed in patients with isolated ARDS due to progressive respiratory failure. None of the patients with isolated ARDS and none of their offspring died. Among the patients with MOF, 2 maternal and 4 fetal deaths occurred. *ARDS* adult respiratory distress syndrome, *MOF* multiorgan failure, *IUFD* intrauterine fetal death

**Fig. 2:**
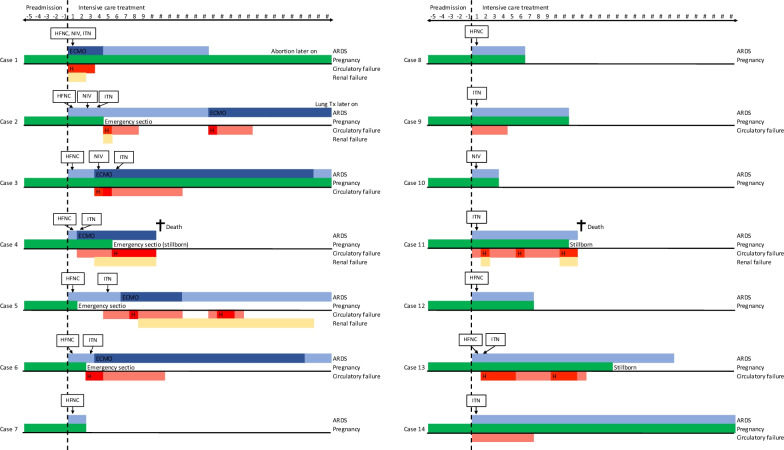
Individual ICU course of included patients. We assessed the use of vasoactive agents for more than 1 day in patients unresponsive to volume challenge as circulatory failure. We distinguished high dose (> 0.1 mcg/kg/min) from low dose catecholamines (< 0.1 mcg/kg/min). Acute kidney injury (AKI) was diagnosed according to the Acute Kidney Injury Network (AKIN) classification. An isolated and marginally elevated bilirubin was not assessed as sign of liver failure and low platelets under ECMO-therapy were not considered to be organ failure, since both had likely other confounders. *HFNC* high flow nasal canula, *NIV* noninvasive ventilation, *ITN* intubation, *ARDS* adult respiratory distress syndrome, *ECMO* extracorporeal membrane oxygenation, *H* high dose catecholamines

Figure 1 summarizes the different ICU courses; Table 1 displays the maternal characteristics. Figure 2 displays the individual ICU course of included patients. The median maternal age was 31 years (Interquartile Range (IQR) 28–37) and the median gestational age on ICU admission 26 weeks (22–32). The median ICU length of stay was 14 days (6–34) days, 13/14 (92.8%) women had severe and 1/14 (12.5%) had moderate ARDS, the median PaO_2_/FiO_2_ (PF ratio) on admission was 74 mmHg (60–93).

**Table 1:** Patient characteristics

	Patient 1	Patient 2	Patient 3	Patient 4	Patient 5	Patient 6	Patient 7	Patient 8	Patient 9	Patient 10	Patient 11	Patient 12	Patient 13	Patient 14
*Maternal factors*														
Age (years)	37	34	38	27	29	30	39	28	32	26	38	28	34	21
Weight (kg)	90	97	70	124	85	87	103	60	90	130	75	72	70	60
BMI	29	34	27	44	32	33	39	23	35	42	27	29	26	22
Gravida/Para	G6/P5	G4/P3	G4/P3	G1/P0	G2/P1	G1/P0	G2/P1	G1/P0	G5/P4	G4/P1	G8/P3	G1/P0	G3/P2	G2/P1
Gestational age (admission ICU)	26	34	22	28	38	33	22	28	24	19	21	31	17	26
Comorbidities	Eclampsia	HIV, hepatitis B		Obesitas	Asthma	Thalassemia	Obesitas			Arterial hypertension	Diabetes, arterial hypertension		Pyelonephritis	
Days on ICU	16	64	52	9	33	38	2	5	10	2	11	6	23	21
*Maternal Covid-19*														
Symptom onset (days)	8	6	4	8	9	7	5	10	9	n.a	8	10	7	n.a
Covid-19 diagNosis	PCR	PCR	PCR	PCR	PCR	PCR	PCR	PCR	PCR	PCR	PCR	PCR	PCR	PCR
CRP (mg/l)	172	115	135	88	119	60	31	138	109	75	174	60	65	184
PCT (mcg/l)	0.2	0.2	0.2	0.2	0.6	0.7	0.2	0.3	0.1	0.1	0.5	0.8	0.8	0.6
White-cell count (× 10^−3^/mm^3^)	21.7	9.2	7.8	7.2	10.7	13.1	7.2	11.8	7.2	7.5	11.8	9.8	7.3	14.2
LDH (U/l)	435	299	379	569	425	464	195	408	393	364	351	310	462	432
Troponin (ng/l)	29	6	< 3.3	n.a	4	5	4	4	9	n.a	10	4	< 3.3	5
Ferritin (mcg/l)	193	76	94	337	179	201	43	99	151	221	171	105	749	290
D-Dimer (mg/l)	3.33	2.01	1.23	2.26	1.69	2.63	1.58	2.5	0.59	0.69	1.81	6.39	0.82	1.02
Fibrinogen (g/l)	6.98	5.82	6.5	6.1	n.a	3.9	n.a	n.a	5.79	6.13	n.a	3.73	n.a	n.a
Invasive ventilation	Yes	Yes	Yes	Yes	Yes	Yes	No	No	Yes	No	Yes	No	Yes	Yes
PEEP/plateau (cm/H_2_O)	16/32	10/26	12/27	15/13	15/16	15/15	n.a	n.a	16/18	n.a	16/15	n.a	12/16	14/16
Horowitz/PF ratio	78	61	62	48	56	70	96	95	112	78	31	67	84	92
Prone positioning during pregnancy	No	No	Yes	Yes	No	No	No	No	Yes	No	Yes	No	Yes	No
ECMO	Yes	Yes	Yes	Yes	Yes	Yes	No	No	No	No	No	No	No	No
Covid-19 targeted therapy	Remdesivir	No	Tocilizumab	No	Tocilizumab	Tocilizumab	No	No	No	Tocilizumab	No	Remdesivir	Remdesivir	Tocilizumab
Systemic steroids	No	Yes	Yes	Yes	Yes	Yes	Yes	Yes	Yes	Yes	Yes	Yes	No	Yes
Vasoactives	Yes	Yes	Yes	Yes	Yes	Yes	No	No	Yes	No	Yes	No	Yes	Yes
AKI	Yes	Yes	No	Yes	Yes	No	No	No	No	No	Yes	No	No	No
Dialysis	Yes	No	No	Yes	Yes	No	No	No	No	No	Yes	No	No	No
Heart failure	No	Yes	No	Yes	Yes	No	No	No	No	No	Yes	No	No	No
SOFA score ad admission	9	3	2	2	2	2	2	2	7	5	^8^	2	3	3
Maternal survival to hospital discharge	Yes	Yes	Yes	No	Yes	Yes	Yes	Yes	Yes	Yes	No	Yes	Yes	Yes
Unborn/newborn survival to hospital discharge	No	Yes	Yes	No	Yes	Yes	Yes	Yes	Yes	Yes	No	Yes	No	Yes
Abortion/stillborn	Yes	No	No	Yes	No	No	No	No	No	No	Yes	No	Yes	No
Delivery during ICU	No	C-section	C-section	C-section	C-section	C-section	No	No	No	No	Transvaginal	No	Transvaginal	No

Displayed are both demographic and clinical patient characteristics of individual patients. Laboratory values and numerical indices of disease severity were recorded at critical care admission

*BMI* body mass index, *G* gravida, *P* para, *ICU* intensive care unit, *CRP* c-reactive protein, *PCT* procalcitonin, *LDH* lactate dehydrogenase, *PEEP* positive endexpiratory pressure, *PF ratio* PaO_2_/FiO_2_ ratio, *ECMO* extracorporeal membrane oxygenation, *AKI* acute kidney injury, *SOFA* sequential organ failure assessment, *MOF* multiorgan failure, *ARDS* adult respiratory distress syndrome, *C-section* cesarean section

10/14 (71.4%) women required invasive mechanical ventilation, 6/14 (42.8%) with additional extracorporeal membrane oxygenation (ECMO). 4/14 (28.5%) patients could be managed with non-invasive support, 3/14 (21.4%) with high flow nasal cannula (HFNC) and 1/14 (7.1%) with non-invasive ventilation (NIV). Prone positioning was used in 5/14 (35.7%) patients. Specific Covid-19 therapies included Remdesivir in 3/14 (21.4%), Tocilizumab in 5/14 (35.7%) and Glucocorticoids in 12/14 (85.7%).

7/14 (50%) women had isolated ARDS in pregnancy and another 7/14 (50%) had multi organ failure (MOF), defined by additional non-pulmonary organ specific sub-SOFA scores ≥ 2 points. In 3/14 (21.4%) MOF developed after delivery of women with previously isolated ARDS.

Considering all MOF together, the second most common organ failure besides ARDS was circulatory failure in 10/14 (71%) women. Kidney failure was present in 5/14 (36%) women. In 4/14 (29%) there was maternal cardiac failure, 3/14 (21.4%) with predominant left heart and one right heart failure, and 2/14 (14.2%) required additional arterial ECMO cannulation for circulatory support.

None of the 7/14 (50%) patients with isolated ARDS during pregnancy died. In 3/14 (21.4%) women, caesarean section was performed while on the ICU between gestational weeks 33 and 38 due to progressive respiratory failure. These women and their offspring survived but all 3 women developed MOF after delivery. All maternal and fetal deaths occurred in patients with MOF who required high-dose catecholamine support: 2/14 (14.2%) of the women and 4/14 (28.5%) of the unborn died. Two intrauterine fetal deaths (IUFD) occurred in the setting of maternal MOF at 21 and 28 weeks’ gestation, respectively. One stillbirth occurred at gestational week 17 after maternal recovery from MOF, and one patient requested abortion at 30 weeks’ gestation after she had already left ICU because her child displayed severe ischemic brain damage presumably resulting from maternal MOF and profound shock.

All 7/14 (50%) women with MOF were before 28 weeks’ gestation, 3/14 (21.4%) were before gestational week 24, before viability, thus delivery was not a reasonable option. The other 4/14 (28.5%) patients with MOF were between gestational week 26 and 28. In these patients, emergency caesarean section was discussed on a daily basis within a multidisciplinary team consisting of critical care and obstetric professionals.

In summary, the management of pregnant patients with severe Covid 19 is complex and requires a multidisciplinary approach. Despite the relatively small sample size, our data suggest that patients with severe Covid-19-related ARDS can be successfully carried through pregnancy with invasive ventilation and ECMO, if needed, as long as they suffer from isolated lung failure. However, the risk of maternal and fetal death increases substantially once MOF develops. Additional circulatory failure requiring high-dose catecholamine support seems to be the major determinant of adverse maternal and fetal outcome in pregnant women with severe Covid-19 associated ARDS.

The decision regarding delivery in women with severe Covid-19 associated ARDS needs to balance multiple risks and benefits, including the risk of prematurity to the fetus, the potential to improve or worsen maternal respiratory status with delivery, and the risks accompanying major surgery such as cesarean section, particularly in patients requiring ECMO support. These preliminary observations need to be tested in larger multicenter studies.

## Data Availability

The datasets used and analyzed during the current study are available from the corresponding author on reasonable request.

## References

[1] Kayem G, Lecarpentier E, Deruelle P, Bretelle F, Azria E, Blanc J, Bohec C, Bornes M, Ceccaldi PF, Chalet Y, Chauleur C, Cordier AG, Desbriere R, Doret M, Dreyfus M, Driessen M, Fermaut M, Gallot D, Garabedian C, Huissoud C, Luton D, Morel O, Perrotin F, Picone O, Rozenberg P, Sentilhes L, Sroussi J, Vayssiere C, Verspyck E, Vivanti AJ, Winer N, Alessandrini V, Schmitz T (2020). A snapshot of the Covid-19 pandemic among pregnant women in France. J Gynecol Obstet Hum Reprod.

[2] (CDC) CfDCaP. Data on COVID-19 during pregnancy. 2022. https://www.cdc.gov/coronavirus/2019-ncov/cases-updates/special-populations/pregnancy-data-on-covid-19.html.

[3] Schnettler WT, Al Ahwel Y, Suhag A (2020). Severe acute respiratory distress syndrome in coronavirus disease 2019-infected pregnancy: obstetric and intensive care considerations. Am J Obstet Gynecol.

[4] Morau E, Bouvet L, Keita H, Vial F, Bonnet MP, Bonnin M, Le Gouez A, Chassard D, Mercier FJ, Benhamou D, Obstetric A, Critical Care Club Working G (2020). Anaesthesia and intensive care in obstetrics during the COVID-19 pandemic. Anaesth Crit Care Pain Med..

[5] Zieleskiewicz L, Chantry A, Duclos G, Bourgoin A, Mignon A, Deneux-Tharaux C, Leone M (2016). Intensive care and pregnancy: Epidemiology and general principles of management of obstetrics ICU patients during pregnancy. Anaesth Crit Care Pain Med.

